# Medical Society of the County of Erie

**Published:** 1903-03

**Authors:** Franklin C. Gram


### SOCIETY PROCEEDINGS.

### Medical Society of the County of Erie.

*Eighty-Second Annual Meeting, January 13, 1903.*

Reported by FRANKLIN C. GRAM, M. D., Secretary.

THE president, Dr. W. M. Ward, called the 82d annual meeting of the Medical Society of the County of Erie to order at 10 a.m., Tuesday, January 13, 1903, in the parlors of the Young Men's Christian Association.

The minutes of the semi-annual meeting and of the memorial meeting, held on the death of Dr. Thomas Lothrop, were read and approved.

A communication received from the New York State Nurses's Association was laid on the table.

Dr. William Warren Potter, chairman of the committee on membership, presented the names of the following applicants, entitled to practise medicine under the laws of this state, and on his motion they were elected members, viz.: Dr. Henry E. Long, Dr. Edward James Cook and Dr. Albert Satterlee.

Dr. W. C. Callanan, librarian, stated that a very substantial gift had been added to the society's library, as shown by a letter from Dr. J. W. Grosvenor, which he submitted. In the letter Dr. Grosvenor stated that the family of the late Dr. John Cole, of Buffalo, had requested him to deliver to the Medical Society of the County of Erie Dr. Cole's library, consisting of 139 volumes. Dr. Grosvenor had complied with the request and had filed a list of the books with the librarian. The cartage charge on the books was $1.50, and on motion of Dr. Callanan the treasurer was directed to reimburse Dr. Grosvenor for his outlay.

Dr. WM. C. CALLANAN urged the society to take more interest in attending the obsequies of deceased members, stating that on some recent occasions the number had been almost disgracefully small.

The president appointed Drs. Fronczak, Stoddart and Jolls a committee to nominate officers for the ensuing year. They submitted the following nominations and on motion the secretary was directed to cast the ballot of the society for the nominees, and they were declared duly elected—namely: president, Ernest Wende; vice-president, John D. McPherson, Akron; secretary, Franklin C. Gram; treasurer, Edward Clark; librarian, Wm. C. Callanan; censors, Irving W. Potter, chairman; John B. Coakley, Henry R. Hopkins, H. Lapp and F. E. Fronczak; committee on legislation, Ernest Wende, William Warren Potter and Edward Clark; chairman of committee on membership, William Warren Potter.

On motion of Wm. C. Krauss the president and secretary were empowered to fill any vacancies that might occur.

Dr. Coakley submitted the following report from the board of censors:

*To the Medical Society of the County of Erie:*

Since its last report your board of censors has continued its work of investigation of cases of alleged illegal practitioners of medicine and surgery in the County of Erie.

On comparing the list of physicians and surgeons of the city as given by the city directory of 1902 with the list contained in the directory of 1901, we find 102 additional names. Each of these 102 persons have been interviewed, either in person or communicated with by circular, and as a result of the information obtained we are enabled to classify them as follows: medical students, 4; graduates of medicine, who failed to pass the examination of the State Board of Medical Examiners, 4; physicians lawfully registered in the county clerk's office, 64; osteopathists, 7; dentists, 9; dental student, 1; persons who had left the city, 5; persons not practising or attempting to practise medicine, 2; United States Marine Hospital Service, 1; electrician, 1; internes in hospital, 2; druggist and chemist, 1; deceased, 1.

We might mention that the censors have called the attention of the parties who have charge of compiling of the city directory to the necessity of care in the classification of physicians and surgeons, and hope that in the future greater care will be taken in eliminating from the list held out to the public by the city directory as physicians and surgeons the names of those who are not lawfully entitled to be classified as such, to-wit: dentists, chemists and other similar classes.

Your board has also called the attention of the medical graduates who have failed to pass their examinations before the state medical board, to the fact that they were not entitled to practise medicine or make use of the title of Dr., and that until they were enabled to pass such examination they should confine themselves strictly to their studies, which the parties above mentioned as having been classified in the directory as physicians and surgeons have consented to do. Internes in the hospitals who appear in the directory as physicians and surgeons have also been warned of the law regulating the practice of the profession and the necessity of their refraining from illegal practice.

There has also been reported to your board four dermatologists, one optician and five chiropodists who were alleged to be illegally practising medicine or at least illegally making use of the title Dr., in their advertising matter. Each of these parties were interviewed by your board or the chairman thereof, notified of the provisions of the law relating to the practice of medicine and warned that they must take down their signs in which they make use of the term Dr. and cease advertising by such title, which in each instance they promptly did.

Dr. F. Gernell Battner, who is or was associated with Dr. Porter, with offices at 333 Main Street, has been reported as illegally practising medicine. The chairman visited Dr. Battner and warned him of the penalty for such illegal practice and he agreed immediately to cease, which the board believes he has done.

Another case which was reported was that of Dr. Leon Smith, with offices in the Lewis Block at the corner of Swan and Washington Streets. He was called upon and warned that until he was duly registered and lawfully entitled to practise in the city, he must remove his sign and cease to make use of the title Dr. or practice the medical profession in the city. Shortly after he took down his sign and removed from the city.

For the past twelve months, your board has made diligent efforts, in season and out of season, to obtain an indictment against a notorious individual with offices on Main Street in this city, near Chippewa, who has been illegally practising medicine. By means of his extensive advertising in the newspapers and the subterfuge of the employment of duly licensed physicians who were so wanting in a sense of manhood and the dignity of their profession as to prostitute the same to their personal profit, he has been enabled to take advantage of the ignorant and credulous and by means of extravagant promises of wonderful and impossible cures to obtain from them large sums of money in the aggregate. Such a condition of affairs in our city is not only a menace to the public health but is an insult to the intelligence of the public. The late District Attorney and his staff, either through indifference, want of confidence in the evidence, or for other reasons not known to this board, refused to present to the various grand juries which have met during the past year the facts which we have submitted, or to advise any indictment of said individual, although in the opinion of this board there has been the very strongest possible evidence against him. It is our earnest hope and expectation that the present administration of the District Attorney's office will realize the menace to the public health which such practises involve and the duty which the district attorney and his associates as public officials owe to the public in protecting it from such violations of law.

Dr. C. H. Woodard, a member of this society, with offices at 850 West Avenue, has been reported to the board as guilty of practises which are in violation of the usages of our profession —first, in his manner of advertising, and, second, in dispensing a secret nostrum. We have interviewed Dr. Woodard and called his attention to his methods as being in violation of the ethics of our profession and requested him to desist therefrom. However, he has declined so to do, claiming that he was justified in all his practises. It is unfortunate for our profession that any of its members are so lacking in a sense of the dignity and honor of the profession that they are willing to seem to prostitute it to their personal ends and will even resort to questionable practises to reach the thoughtless und unfortunate. It is probable that there is no legal method to prevent such practises and that our only redress therefor is expulsion of the offender from fellowship in this society.

In conclusion, this board begs to acknowledge its indebtedness to its counsel, Hon. Tracy C. Becker, for valuable assistance rendered to it in the past and would respectfully recommend that he be continued as counsel for the board, and that the board be given authority to employ such detectives and other agents as may be required for the performance of its duties, and to draw upon the treasurer for such reasonable compensation for its counsel and disbursements as the board may approve.

Respectfully submitted,

J. B. Coakley, Chairman.
Irving W. Potter,
Francis E. Fronczak.
H. R. Hopkins.
Henry Lapp.

Dated January 13, 1903.

The report was adopted and on motion the president appointed John H. Grant, C. C. Wyckoff and William Warren Potter a committee to investigate the Woodard case.

The treasurer, Dr. Edward Clark, submitted his annual report, which was referred to an auditing committee, consisting of Drs. Hourigan and Grant. The committee subsequently reported the accounts of the treasurer to be correct.

On motion of Dr. Callanan, the treasurer was empowered to employ such means as in his discretion may become necessary for the collection of membership dues.

The scientific program was then taken up. Hon. C. D. Davie, of Salamanca, N. Y., Surrogate of Cattaraugus County, read a paper on Medical Expert Testimony (See page 566). The discussion was participated in by Hon. Tracy C. Becker and Clarence M. Bushnell, Esq., on the part of the legal profession and Drs. Floyd S. Crego, Wm. C. Krauss and James W. Putnam on the part of the medical profession.

The attendance numbered over 50 members together with several guests. Among the latter was Dr. A. D. Lake, of Gowanda.

The society at 1.30 p. m. adjourned.

